# Absence of CD28-CTLA4-PD-L1 Costimulatory Molecules Reduces Herpes Simplex Virus 1 Reactivation

**DOI:** 10.1128/mBio.01176-21

**Published:** 2021-07-20

**Authors:** Harry H. Matundan, Ujjaldeep Jaggi, Jack Yu, Omid Akbari, Homayon Ghiasi

**Affiliations:** a Center for Neurobiology and Vaccine Development, Ophthalmology Research, Department of Surgery, Cedars-Sinai Burns & Allen Research Institute, Los Angeles, California, USA; b Department of Molecular Microbiology and Immunology, Keck School of Medicine, University of Southern California, Los Angeles, California, USA; Princeton University

**Keywords:** HSV-1, antigen-presenting cells, corneal scarring, knockout, latency, ocular infection, reactivation

## Abstract

We previously reported that herpes simplex virus 1 (HSV-1) ICP22 binds to CD80 and suppresses CD80 expression *in vitro* and *in vivo*. Similar to ICP22, the cellular costimulatory molecules CD28, CTLA4, and PD-L1 also bind to CD80. In this study, we asked whether, similar to ICP22-null virus, the absence of these costimulatory molecules will reduce HSV-1 infectivity. To test our hypothesis, CD28^−/−^, CD28^−/−^ CTLA4^−/−^, PD-L1^−/−^, and wild-type control BALB/c mice were ocularly infected with HSV-1 strain KOS. Levels of virus replication in the eye, corneal scarring (CS), latency, and reactivation in infected mice were determined. Expression of different genes in the trigeminal ganglia (TG) of latently infected mice was also determined by NanoString and quantitative reverse transcription-PCR (qRT-PCR). In the absence of costimulatory molecules, latency levels were higher than those in wild-type control mice, but despite higher latency, a significant number of TG from infected knockout mice did not reactivate. Reduced reactivation correlated with downregulation of 26 similar cellular genes that are associated with inflammatory signaling and innate immune responses. These results suggest that lower reactivation directly correlates with lower expression of interferon signaling. Thus, despite having different modes of actions, we identified a similar function for CD28, CTLA4, and PD-L1 in HSV-1 reactivation that is dependent on their interactions with CD80. Therefore, blocking these interactions could be a therapeutic target for HSV-1-induced reactivation.

## INTRODUCTION

Previously, we have shown that herpes simplex virus 1 (HSV-1) ICP22 downregulates expression of CD80 but not CD86 independent of the presence of anti-HSV-1 antibody (1). Suppression of CD80 by ICP22 is mediated by direct binding of HSV-1 ICP22 to CD80 (1). CD80 plays a critical role in increased inflammatory responses in HSV-1 infected mice corneas (2, 3), and we have also shown that a recombinant HSV-1 that overexpresses CD80 exacerbates corneal scarring (CS) in infected BALB/c and C57BL/6 mice (1, 4). Further, mice ocularly infected with a recombinant HSV-1 lacking ICP22 developed enhanced eye disease despite the reduced infectivity of this virus compared with parental virus (3). Our published studies have also shown that expression of CD80 by HSV-1 in place of LAT compensated for the latency-reactivation and antiapoptotic functions of LAT (4). Thus, the ability of HSV-1 to suppress CD80 expression requires the HSV-1 ICP22 gene, and suppression of CD80 by ICP22 is a mechanism of virus self-preservation.

Binding of CD28 on the surface of T cells to CD80 on the surface of antigen-presenting cells is required for T cell activation, and this interaction leads to T cell proliferation, differentiation, and cytokine secretion (5, 6). Similar to CD28, CTLA4 also binds to CD80, and this interaction blocks the ability of CD28 to bind CD80 (7). CD80 is expressed on a variety of cells, including B cells, macrophages, dendritic cells, and T cells (8–11). In addition to CD80 and CD86, B7 pathways include the PD-1 receptor and its two ligands, PD-L1 and PD-L2 (5, 12, 13). Similar to CD28 and CTLA4, PD-L1 (but not PD-L2) binds to CD80 and inhibits T cell proliferation and cytokine production (14, 15).

The expression of CD80 or CD86 is inducible by gamma interferon (IFN-γ) in nonlymphoid tissues (5, 16, 17). In B cells, CD80 elicits a negative signal for proliferation and inhibits IgG secretion, while CD86 can increase the activity of B cells (18). Engagement of CD28 with its ligand leads to activation of naive T cells and T cell proliferation with interleukin-2 (IL-2) production (17). The CTLA4 receptor is rapidly expressed in T cells after activation but not in B cells or myeloid cells. CTLA4 acts as an immune brake and inhibits T cell proliferation and IL-2 production, and other cytokine production and its absence in mice leads to defects in the regulation of T-cell proliferation and T_H_ cytokine production (5). Inhibition of T cell activation with the fusion protein CTLA4Ig abrogated HSK in ocularly infected mice (19). Other important mediators of immune response are PD-L1 and PD-L2 and their immune receptor, PD-1 (20). The PD-1 receptor is expressed shortly after activation in T cells, B cells, and myeloid cells. PD-L1 also is expressed on T cells, B cells, monocytes, and dendritic cells (DCs), while PD-L2 is found on DCs, splenic tissue, and lymph nodes. PD-1 activation results in function similar to that of CTLA4, in that it acts as an immune regulator that inhibits T-cell and myeloid cell proliferation as well as effector cytokine production (5, 17). Previously, we have shown that the absence of PD-1 and PD-L1 but not PD-L2 significantly reduced latency in ocularly infected mice (21). However, very little is known about the role, if any, of CD28, CTLA4, and PD-L1 binding to CD80 in HSV-1 infectivity *in vivo*.

Since HSV-1 uses CD80 suppression as a means of self-survival, in this study we evaluated the effect of CD80 interactions with CD28, CTLA4, and PD-L1 during primary and latent HSV-1 infection in ocularly infected mice. We used CD28^−/−^, CD28^−/−^ CTLA4^−/−^, and PD-L1^−/−^ mice to determine possible roles of the CD28-CTLA4-PD-L1 axis and their lack of interaction with CD80 in primary ocular infection, eye disease, and latency reactivation following infection with wild-type HSV-1. In the absence of CD28, CTLA4, or PD-L1, viral titers were higher in the eyes of infected mice than in wild-type (WT) mice. In addition, latency levels were also higher in the absence of CD28, CTLA4, or PD-L1 than in WT control mice. However, despite higher virus replication in the eye and higher latency in the TG, the absence of CD28, CD28-CTLA4, or PD-L1 significantly reduced reactivation in ocularly infected mice. Our results suggest that interaction of CD28, CTLA4, or PD-L1 with CD80 is required for efficient reactivation. Mechanisms that increase reactivation are of great clinical significance because critical eye disease occurs following HSV-1 reactivation. Indeed, a major cause of eye disease is the corneal scarring induced by HSV-1 following reactivation from latency (22–24). Overall, these costimulatory pathways, despite having different modes of action, played similar roles with regard to primary infection, establishment of latency, and reactivation from latency. Thus, our studies demonstrate that the binding of CD80 to CD28, CTLA4, or PD-L1 plays a vital role in faster reactivation of HSV-1 and that blocking this interaction reduces reactivation and, thus, immune-mediated pathology associated with viral reactivation.

## RESULTS

### Absence of CD28, CTLA4, or PD-L1 increases HSV-1 replication in mouse eyes.

**FIG 1:**
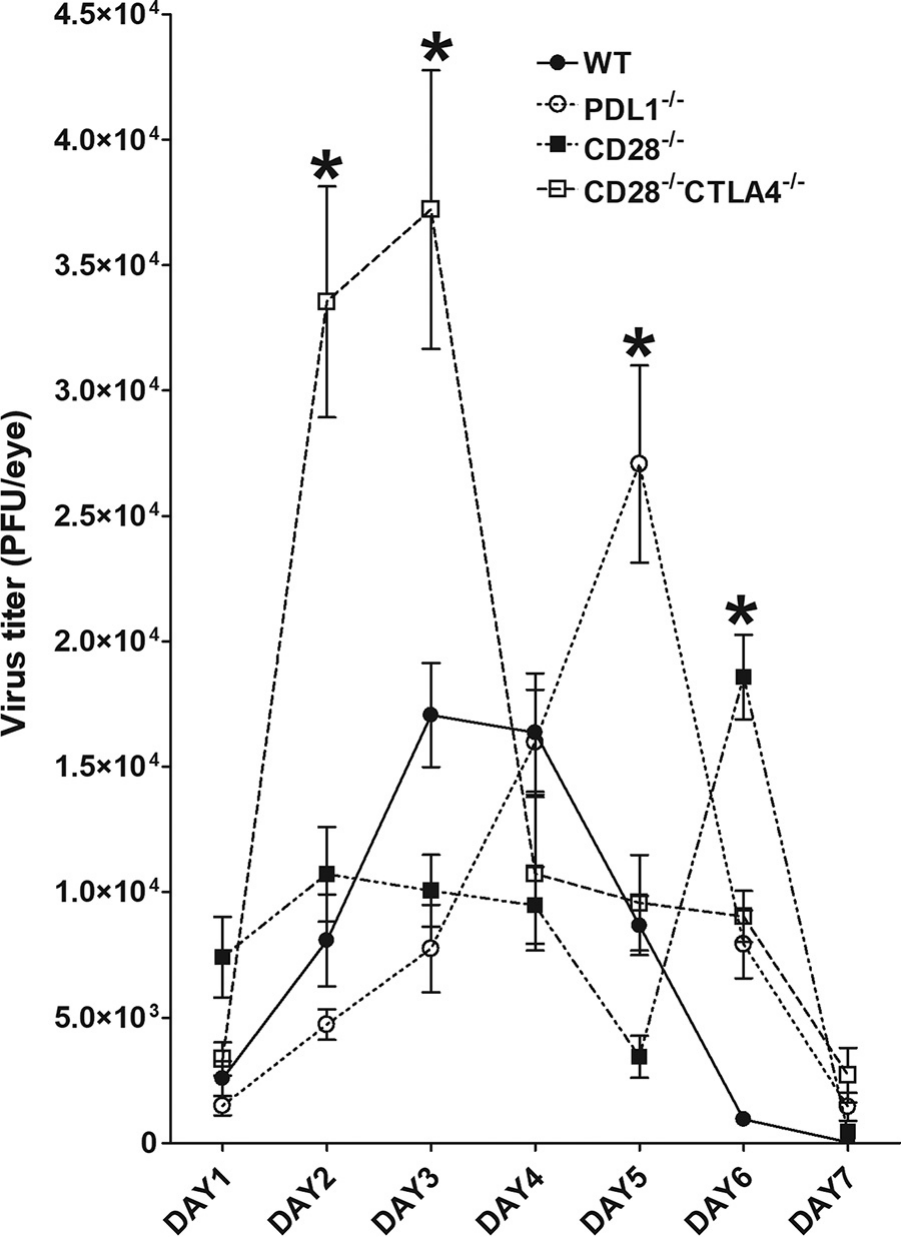
Viral titers in infected mouse eyes following ocular HSV-1 infection. Corneas from CD28^−/−^, CD28^−/−^ CTLA4^−/−^, PD-L1^−/−^, and WT control mice were scarified before ocular infection. Mice were then infected ocularly with 2 × 10^5^ PFU per eye of avirulent KOS strain of HSV-1. Presence of infectious virus in the eyes of infected mice was monitored daily for 7 days by collecting tear films and quantifying virus using standard plaque assays as described (see Materials and Methods). Each point represents the means ± SEM for CD28^−/−^ mice (30 eyes), CD28^−/−^ CTLA4^−/−^ mice (36 eyes), PD-L1^−/−^ mice (32 eyes), and WT mice (30 eyes).

We previously showed that CD80 expression is downregulated by HSV-1 infection due to HSV-1 ICP22 binding to CD80 (1, 3). Because CD28, CTLA4, and PD-L1 also bind to CD80 (7, 14, 15), we examined the effect of those molecules on HSV-1 infection *in vivo*, using WT, CD28^−/−^, CD28^−/−^ CTLA4^−/−^, and PD-L1^−/−^ mice. Following corneal scarification, each mouse strain was ocularly infected with 2 × 10^5^ PFU/eye of avirulent HSV-1 strain KOS. CD28^−/−^ CTLA4^−/−^ mice were used in this study because CTLA4^−/−^ mice are not viable (25). Tear films were collected from 11 to 18 mice per group on days 1 to 7 postinfection p.i. in 2 to 3 experiments. Virus titers, determined by standard plaque assays as described in Materials and Methods, were similar in all groups on day 1 p.i. (Fig. 1) (*P* > 0.05). On days 2 to 3 p.i., CD28^−/−^ CTLA4^−/−^ infected mice had significantly higher viral titers than WT, CD28^−/−^, or PD-L1^−/−^ mice (Fig. 1) (*P* < 0.05). However, virus titers on day 4 p.i. were similar among the four mouse groups (Fig. 1) (*P* > 0.05). PD-L1^−/−^ mice on day 5 p.i., and CD28^−/−^ mice on day 6 p.i., had significantly higher viral titers than WT and CD28^−/−^ CTLA4^−/−^ mice (Fig. 1) (*P* < 0.05). By day 7 p.i., viral titers had declined and all groups had similar titers (Fig. 1) (*P* > 0.05). These results suggest that the absence of CD28^−/−^, CD28^−/−^ CTLA4^−/−^, or PD-L1^−/−^ significantly increased virus replication in the eyes of infected mice, and the absence of each molecule differentially increased the pattern of viral replication compared to WT control mice.

### Lack of CD28, CTLA4, or PD-L1 did not alter corneal scarring.

**FIG 2:**
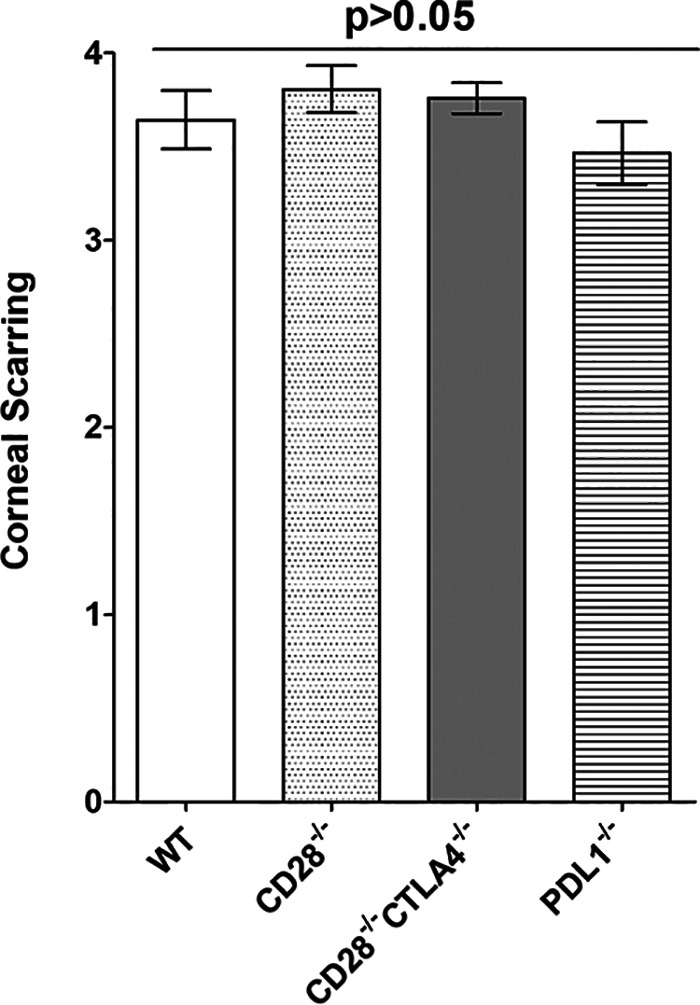
Loss of binding to CD80 does not affect severity of corneal scarring. Corneas from infected CD28^−/−^, CD28^−/−^ CTLA4^−/−^, PD-L1^−/−^, and WT mice described in Fig. 1 were evaluated for severity of CS on day 28 after ocular infection by slit-lamp biomicroscopy. Experiments were repeated three times, and each bar represents the means ± SEM for infected CD28^−/−^ mice (57 eyes), CD28^−/−^ CTLA4^−/−^ mice (54 eyes), PD-L1^−/−^ mice (44 eyes), and WT mice (42 eyes).

The immune response leading to HSV-1-induced CS is a cell-mediated immune response (26). Since T cells have been implicated in CS and CD28, CTLA4, and PD-L1 influence both innate and adaptive immune responses to infection, we asked whether the absence of CD28, CTLA4, or PD-L1 would affect the progression of CS in ocularly infected mice. On day 28 p.i., CS in mice described for Fig. 1 was analyzed, and no significant differences in CS were observed in infected CD28^−/−^, CD28^−/−^ CTLA4^−/−^, PD-L1^−/−^, or WT mice (Fig. 2) (*P* > 0.05). Thus, our results suggest that although these three knockout mice have higher virus replication in their eyes than WT mice, the absence of CD28, CTLA4, or PD-L1 did not alter viral pathology compared to WT infected mice.

### Absence of CD28, CTLA4, and PD-L1 costimulatory molecules enhanced latency in latently infected mice.

**FIG 3:**
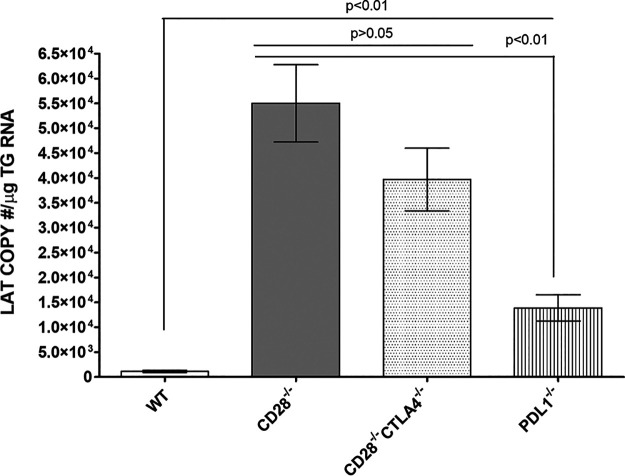
Differential effect of loss binding to CD80 on latency in infected mice. Corneas from CD28^−/−^, CD28^−/−^ CTLA4^−/−^, PD-L1^−/−^, and WT mice were scarified and infected ocularly with 2 × 10^5^ PFU per eye of KOS virus as shown in Fig. 1. On day 28 p.i., TG were harvested from infected mice, and qRT-PCR was performed on individual TG. In each experiment, an estimated relative copy number of HSV-1 LAT was calculated using standard curves generated from pGem5317. The plasmid template was serially diluted 10-fold such that 10 μl contained 10^3^ to 10^11^ copies of LAT. Serial dilutions were then analyzed by TaqMan real-time PCR with the same probe set. The copy number for each reaction was determined by comparing the normalized threshold cycle of each sample to the threshold cycle of the standard. GAPDH expression was used to normalize relative viral LAT RNA expression in the TG. Experiments were repeated three times, and each bar represents the means ± SEM from for CD28^−/−^ infected mice (39 TG), CD28^−/−^ CTLA4^−/−^ infected mice (34 TG), PD-L1^−/−^ infected mice (34 TG), and WT infected mice (36 TG).

Since establishing latency is a major characteristic of HSV-1 infection and is mediated by immune cells (21, 27), we asked how the absence of CD28, CTLA4, or PD-L1 binding to CD80 would affect latency establishment. CD28^−/−^, CD28^−/−^ CTLA4^−/−^, PD-L1^−/−^, and WT control mice were infected with HSV-1 strain KOS as described for Fig. 1. Previously, we reported a direct correlation between the level of HSV-1 LAT RNA and the level of HSV-1 gB DNA (21); thus, on day 28 p.i., TG from infected mice were isolated, and LAT transcript levels in latently infected individual TG were measured by quantitative reverse transcription-PCR (qRT-PCR). Significantly higher levels of LAT mRNA were detected in the TG of both CD28^−/−^ and CD28^−/−^ CTLA4^−/−^ infected mice than in TG from WT or PD-L1^−/−^ mice (Fig. 3) (*P* < 0.01, CD28^−/−^ and CD28^−/−^ CTLA4^−/−^ infected mice versus WT and PD-L1^−/−^ infected mice). However, levels of latency were similar in both CD28^−/−^ and CD28^−/−^ CTLA4^−/−^ infected mice (Fig. 3) (*P* > 0.05). Finally, PD-L1^−/−^ infected mice had significantly higher levels of latency than did WT mice (Fig. 3) (*P* < 0.01). Overall, the absence of CD28, CTLA4, and PD-L1 significantly increased latency in infected mice compared with infected WT mice. This negative effect was more pronounced in the absence of CD28 and CTLA4 than in the absence of PD-L1. Thus, in these three knockout mice, levels of latency correlated positively with primary virus replication in the eye.

### Absence of CD28, CTLA4, and PD-L1 delayed/prevented virus reactivation in TG of latently infected mice.

**FIG 4:**
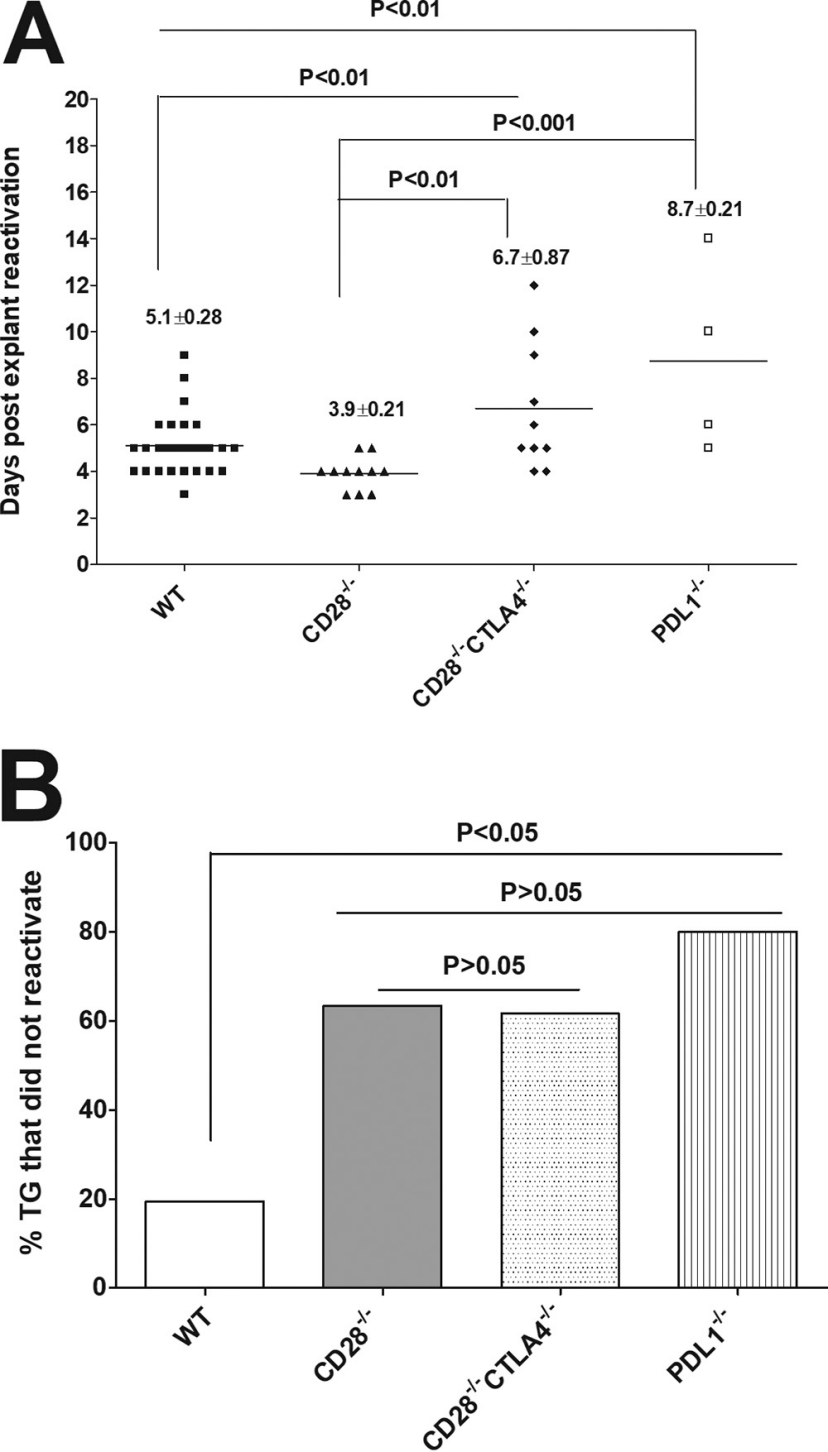
Duration and lack of reactivation is affected in the absence of CD28, CTLA4, and PD-L1 in infected mice. Corneas from CD28^−/−^, CD28^−/−^ CTLA4^−/−^, PD-L1^−/−^, and WT mice were scarified before ocular infection and infected ocularly with 2 × 10^5^ PFU per eye of KOS virus as described for Fig. 1. On day 28 p.i., TG from infected mice were harvested for explant reactivation. (A) Reactivated explant TG. Individual TGs from infected mice were incubated in 1.5 ml of tissue culture medium at 37°C, and the presence of infectious virus was monitored for 20 days as described in Materials and Methods. For CD28^−/−^ and CD28^−/−^ CTLA4^−/−^ infected mice, 34 TG from 17 mice were used. For PD-L1^−/−^ infected mice, 20 TG from 10 mice were used, and 38 TG from 19 mice were used for WT mice. The results are shown as the number of TG that reactivated daily. Each point represents the mean ± SEM from infected CD28^−/−^ mice (11 TG), CD28^−/−^ CTLA4^−/−^ mice (10 TG), PD-L1^−/−^ mice (4 TG), and WT mice (31 TG). (B) Percentage of TG that did not reactivate after 20 days of explant. Not all TG reactivated during the 20-day monitoring period. In CD28^−/−^ infected mice, 23/34 TG did not reactivate; in CD28^−/−^ CTLA4^−/−^ infected mice, 24/34 TG did not reactivate; in PD-L1^−/−^ infected mice, 16/20 TG did not reactivate; and in WT infected mice, 7/38 TG did not reactivate. The numbers of TG that did not reactivate are shown as percentages of total TG used per mouse strain. (A) Number of reactivated explant TG. (B) Percentage of explant TG that were not reactivated.

To determine whether increased LAT expression in TG of CD28^−/−^, CD28^−/−^ CTLA4^−/−^, and PD-L1^−/−^ mice correlated with faster reactivation of latent virus, mice were ocularly infected as described for Fig. 1 with HSV-1 strain KOS. On day 28 p.i., latently infected TG were harvested from 10 to 19 mice per group in 2 to 3 experiments, and the kinetics of virus reactivation in individual TG was monitored for 20 days in explanted TG, as we described previously (28). The number of TG that were reactivated by day 20 postexplant culture (Fig. 4A) and the percent TG per group that were not reactivated after 20 days (Fig. 4B) are shown. We observed significant delays in reactivation of infected CD28^−/−^ CTLA4^−/−^ (6.7 days) and PD-L1^−/−^ (8.7 days) mice compared to WT infected mice (5.1 days) and CD28^−/−^ infected mice (3.9 days) (Fig. 4A). Reactivation levels in WT and CD28^−/−^ infected TG groups were statistically different from each other (Fig. 4A) (*P* < 0.05) and from CD28^−/−^ CTLA4^−/−^ and PD-L1^−/−^ infected mice (Fig. 4A) (*P* < 0.001). Thus, TG reactivation in CD28^−/−^ mice was faster than in other groups.

Although TG reactivation in CD28^−/−^ mice was faster than in other groups, the majority of TG in the knockout mouse groups did not reactivate (Fig. 4B). In WT mice, only 19% of infected TG did not reactivate, while in CD28^−/−^, CD28^−/−^ CTLA4^−/−^, and PD-L1^−/−^ infected mice, 63%, 61%, and 80% of TG, respectively, did not reactivate (Fig. 4B). The difference in reactivation of WT infected TG was statistically different from the three knockout groups (Fig. 4B) (*P* < 0.05), while differences between numbers of TG that did not reactivate in CD28^−/−^, CD28^−/−^ CTLA4^−/−^, and PD-L1^−/−^ infected TG were not statistically significant (Fig. 4B) (*P* > 0.05). Thus, in the absence of CD28, CTLA4, or PD-L1, infected mice showed less reactivation than WT infected mice despite having higher levels of latency and more primary virus replication.

### Factors contributing to delayed reactivation.

Significantly more latently infected TG from knockout mice did not reactivate by day 20 than in control WT mice. Thus, we asked why loss of these immune costimulatory receptors delayed reactivation despite having higher virus replication in the eyes and higher levels of latency than control WT mice. We used the nCounter MM Myeloid Innate Immunity V2 panel to identify factors shared between the three strains of mice that may contribute to delayed reactivation. After corneal scarification, CD28^−/−^, CD28^−/−^ CTLA4^−/−^, PD-L1^−/−^, and WT control mice were ocularly infected with 2 × 10^5^ PFU/eye of HSV-1 strain KOS. On day 28 p.i., TG from 36 infected mice (9/mouse strain) were isolated. To reduce variability, RNA collected from three mice per group were combined and analyzed on the nCounter FLEX platform as described in Materials and Methods.

**FIG 5:**
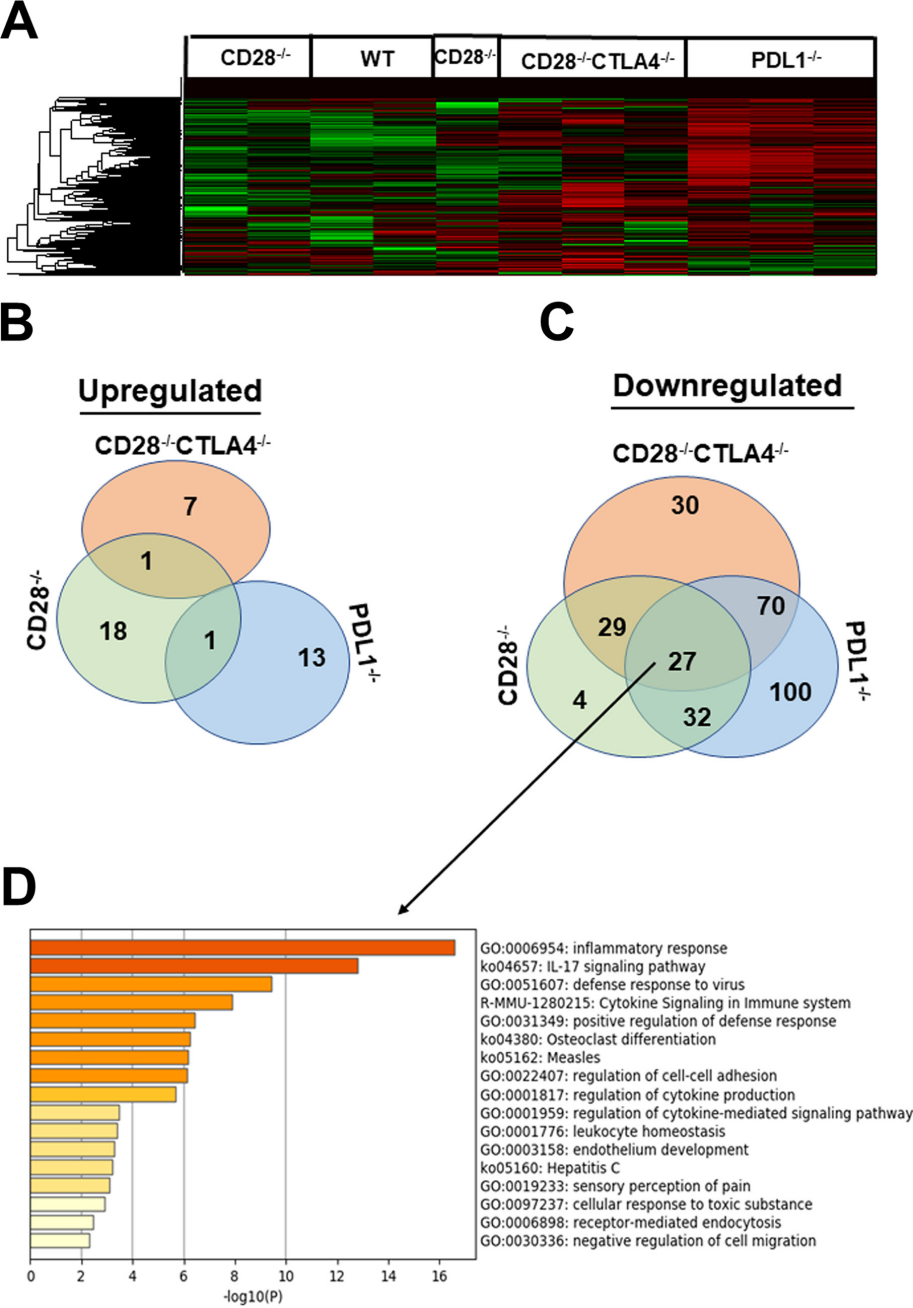
NanoString gene expression analysis of latently infected TG. CD28^−/−^, CD28^−/−^ CTLA4^−/−^, PD-L1^−/−^, and WT mice were ocularly infected with 2 × 10^5^ PFU/eye of HSV-1 strain KOS after corneal scarification. TG from infected mice on day 28 postinfection were isolated, and total RNA from individual TG was isolated. RNA samples for use in the NanoString nCounter sample input were pooled from 3 mice (6 TG), and each experiment was repeated three times using a total of 9 mouse TG (18 TG) for each mouse strain. Total RNA concentration in each well remained at 20 ng/μl. Expression of the 764-gene myeloid immune panel was analyzed as described in Materials and Methods. Differentially expressed genes were identified for each group by normalizing samples to housekeeping genes and infected WT mice. (A) Heatmaps were used to identify genes that were upregulated (green) or downregulated (red), and black displays no change in mRNA expression. Venn diagrams show the numbers of genes in infected CD28^−/−^, CD28^−/−^ CTLA4^−/−^, and PD-L1^−/−^ mice when normalized to infected WT control that are uniquely or commonly upregulated (B) or downregulated (C). The 27 common downregulated genes in CD28^−/−^, CD28^−/−^ CTLA4^−/−^, and PD-L1^−/−^ infected mice (B) were further analyzed using Metscape (D). Based on this analysis, the top five pathways were inflammatory response (18 of the 26 genes), IL-17 signaling (23 of 26 genes), defense response to virus (14 of 26 genes), cytokine signaling, and positive regulation of defense response.

After normalization, unsupervised hierarchal clustering was used to analyze the expression of 764 genes in the myeloid immune panel. Some differences in gene expression patterns of latently infected TG were identified between WT, CD28^−/−^, CD28^−/−^ CTLA4^−/−^, and PD-L1^−/−^ infected mice. The heatmap identified an expression pattern that was visually distinct between infected mice (Fig. 5A). Detailed review of the pattern suggested that CD28^−/−^ CTLA4^−/−^ and PD-L1^−/−^ infected mice shared a pattern that mostly showed downregulated expression of transcripts during latency compared to CD28^−/−^ and WT mice (Fig. 5A). In contrast, CD28^−/−^ and WT mice shared a pattern of gene expression during latency, seen as green in the heatmap, which highlights the activation of specific pathways that characterize their host’s myeloid immune response during HSV-1 latency (Fig. 5A).

Differentially expressed genes were identified in CD28^−/−^, CD28^−/−^ CTLA4^−/−^, and PD-L1^−/−^ mice by normalizing samples to housekeeping genes and infected WT mice. The list of statistically significant upregulated and downregulated genes (Table 1) for each mouse strain was refined by creating a Venn diagram to group shared genes and discriminate them from genes that identified an inflammatory response signature during latent infection (Fig. 5B and C). In CD28^−/−^ infected mice, 18 genes were upregulated compared to WT infected mice: *Hip1r*, *Akap13*, *Mertk*, *Gpr183*, *Lta4h*, *F11r*, *Vav2*, *Fadd*, *Id1*, *Id3*, *Stat4*, *Apoe*, *H2-Ab1*, *Cd74*, *Ltb*, *Cxcr6*, *H2-Q2*, and *Cd8* (Fig. 5B, CD28^−/−^). In CD28^−/−^ CTLA4^−/−^ mice, 7 genes were upregulated compared to WT infected mice (*Vegfc*, *Il9*, *Cldn1*, *Ngf*, *Ptgds*, *Cx3cr1*, and *Siglecf*) (Fig. 5B, CD28^−/−^ CTLA4^−/−^). In PDL-1^−/−^ mice, 13 genes were upregulated compared to WT infected mice (*Cdh4*, *Mapk12*, *Fgf18*, *Adam19*, BclII, *Insig1*, *Tspan8*, *Prkca*, *Nmb*, *Sesn1*, *Serpinb6a*, *Tuba4a*, and *Sptbn1*) (Fig. 5B, PD-L1^−/−^). Rasal1 was the only gene upregulated in both CD28^−/−^ and CD28^−/−^ CTLA4^−/−^ mice. Similarly, Cxcr3 was a common gene upregulated in both CD28^−/−^ and PD-L1^−/−^ infected mice (Fig. 5B).

**TABLE 1:** List of genes significantly upregulated or downregulated in the three knockout mouse lines relative to WT mice^*a*^

CD28^−/−^	CD28^−/−^ CTLA4^−/−^	PDL1^−/−^		
*Ccl4*	*Nod1*	*Tlr9*	*Tlr13*	*Vsir*
*Lif*	*Icos*	*Was*	*Icosl*	*Furin*
*Nr4a2*	*Amica1*	*Il15*	*Top2a*	*Adgre1*
*Klf10*	*Ldlr*	*Arg1*	Fgf18*	*Ifnar2*
Hip1r*	*Gata2*	*Nlrp3*	*Rab20*	*Skil*
Akap13*	*Btg2*	*Alox5*	*Gpr65*	*Fscn1*
Mertk*	*Nectin2*	*Adcyap1r1*	Adam19*	*C3ar1*
Gpr183*	*Ccr9*	*Btla*	*Cytip*	*Adamts1*
Lta4h*	*Flt1*	*Rgs1*	*Gadd45b*	*Cxcl16*
F11r*	*Nfkbie*	*Cd80*	*Irf8*	*Cybb*
Vav2*	*Abcf1*	*Ms4a1*	*Vav1*	*Ptprc*
Fadd*	*Dusp2*	*Ccl3*	*Tlr8*	*Lipa*
Id1*	*Cdh1*	*Klk1*	*Alox5ap*	*Cxcl14*
Id3*	*Il4ra*	*Mmp19*	*Itgam*	*Anxa4*
Stat4*	*Fgfr1*	*Lst1*	*Cd180*	*Grn*
Apoe*	*Traf6*	Cdh4*	*Plau*	*Cxcl9*
H2-Ab1*	*Acly*	*Ccl19*	*Cd38*	*Cd68*
Cd74*	*Foxp3*	*Siglec1*	*Trafd1*	*Laptm5*
Ltb*	*Timp3*	*Plaur*	*Tnfaip8*	*Rhog*
Cxcr6*	*Calr*	Mapk12*	*Cxcl11*	Insig1*
H2-Q2*	*Map2k1*	*Csf2ra*	BclII*	Tspan8*
Cd8*	*Stat6*	*Tnfrsf1b*	*Itgb2*	*Sirpa*
	*Glg1*	*Sema4a*	*Tlr4*	Prkca*
	*Mapk14*	*Gem*	*Clec7a*	Nmb*
	*Map2k2*	*Fpr2*	*Tnfrsf12a*	Sesn1*
	*Tfcp2*	*Tlr2*	*Nfkb1*	*Tyrobp*
	*Lag3*	*Pik3cg*	*Cd84*	*Prdx3*
	*Gnai3*	*Btk*	*Selplg*	*Id2*
	*Coasy*	*Adam8*	*Pdgfra*	Serpinb6a*
	*Msh2*	*Mmp12*	*Trem2*	*Ctss*
	Vegfc*	*Lat2*	*Anxa1*	*Psme2*
	Il9*	*Ptafr*	*Il1rap*	*C1qc*
	Cldn1*	*Tlr3*	*Fcgr3*	*Cstb*
	Ngf*	*Hpgds*	*Cd274*	Tuba4a*
	Ptgds*	*Cd86*	*Rin2*	Sptbn1*
	Cx3cr1*	*Csf3r*	*Pros1*	*H2-D1*
	Siglecf*	*Syk*	*Mafb*	*H2-K1*
		*Cxcr4*	*Marcksl1*	

a Upregulated genes are shown with asterisk.

We also examined the downregulated genes in TG of infected mice relative to WT mice (Fig. 5C). In CD28^−/−^ infected mice, 4 genes were downregulated compared to WT infected mice: *Ccl4*, *Lif*, *Nr4a2*, and *Klf10* (Fig. 5C). In CD28^−/−^ CTLA4^−/−^ mice, 30 genes were downregulated compared to WT infected mice (*Nod1*, *Icos*, *Amica1*, *Ldlr*, *Gata2*, *Btg2*, *Nectin2*, *Ccr9*, *Flt1*, *Nfkbie*, *Abcf1*, *Dusp2*, *Cdh1*, *Il4ra*, *Fgfr1*, *Traf6*, *Acly*, *Foxp3*, *Timp3*, *Calr*, *Map2k1*, *Stat6*, *Glg1*, *Mapk14*, *Map2k2*, *Tfcp2*, *Lag3*, *Gnai3*, *Coasy*, and *Msh2*) (Fig. 5C, Table 1). In contrast to CD28^−/−^ and CD28^−/−^ CTLA4^−/−^ infected mice, up to 100 genes were downregulated in TG of PD-L1^−/−^ infected mice compared to WT infected mice (Table 1). The Venn diagram (Fig. 5C) also shows 29 downregulated genes in common between CD28^−/−^ mice and CD28^−/−^ CTLA^−/−^ mice. In comparison, 32 genes were downregulated in both CD28^−/−^ and PD-L1^−/−^ infected mice (Fig. 5C). Seventy genes were downregulated in both CD28^−/−^ CTLA4^−/−^ and PD-L1^−/−^ mice (Fig. 5C). Finally, 27 genes (26 cellular genes and 1 HSV-1 ICP22 gene) were significantly downregulated in CD28^−/−^, CD28^−/−^ CTLA4^−/−^, and PD-L1^−/−^ infected mice compared to WT infected mice (Fig. 5C, Table 2). Thus, among the 764 genes analyzed, only 27 genes were commonly downregulated in all three knockout mice. One of the 27 downregulated genes was ICP22, and the other 26 common downregulated cellular genes in CD28^−/−^, CD28^−/−^ CTLA4^−/−^, and PD-L1^−/−^ infected mice were further analyzed using Metascape (Fig. 5D). The top five pathways identified in this analysis were inflammatory response, IL-17 signaling, defense response to virus, cytokine signaling in the immune system, and positive regulation of defense response pathways. The inflammatory response pathway contained 18 of the 26 genes, the IL-17 signaling pathway contained 23 of the 26 genes, and the defense response to virus pathway contained 14 of 26 genes. Thus, most of the 26 common genes between the three knockout mice are related to inflammation and innate immune responses.

**TABLE 2:** List of 27 common downregulated genes in the three knockout mouse lines relative to WT mice^*a*^

Gene designation	Level of downregulation by mouse strain		
	CD28^−/−^	CD28^−/−^ CTLA4^−/−^	PDL1^−/−^
*Ccl12*	−3.76	−3.34	−4.22
*Ccl2*	−4.13	−5.33	−8.05
*Ccl7*	−4.65	−5.61	−7.09
*Ccrl2*	−1.41	−1.82	−2.95
*Cd25*	−1.71	−3.20	−3.19
*Cd40*	−2.01	−4.93	−4.10
*Cebpb*	−1.41	−1.58	−1.87
*Clic4*	−1.49	−1.75	−1.98
*Cxcl1*	−1.98	−2.88	−2.88
*Cxcl10*	−6.76	−11.73	−9.85
*Daxx*	−2.49	−3.04	−2.73
*Fcgr1*	−1.94	−2.20	−3.34
*Fcgr4*	−1.62	−1.51	−2.35
ICP22 (HSV-1)	−3.07	−3.07	−3.07
*Ifit1bl1*	−4.06	−4.91	−5.85
*Il1β*	−1.86	−3.26	−2.48
*Il1rn*	−4.63	−7.98	−8.89
*Irf2*	−1.28	−1.42	−1.60
*Irf7*	−4.79	−9.14	−10.46
*Isg15*	−5.24	−5.46	−6.44
*Mx1*	−2.69	−3.11	−3.04
*Mx2*	−4.30	−5.00	−4.89
*Nampt*	−1.32	−1.52	−2.04
*Socs1*	−2.65	−3.91	−3.88
*Socs3*	−1.78	−2.66	−2.16
*Tnfaip3*	−1.29	−1.77	−2.38
*Usp18*	−4.09	−5.44	−6.05

a Mean for each gene was normalized to the mean of WT infected mice. *n* = 3.

**FIG 6:**
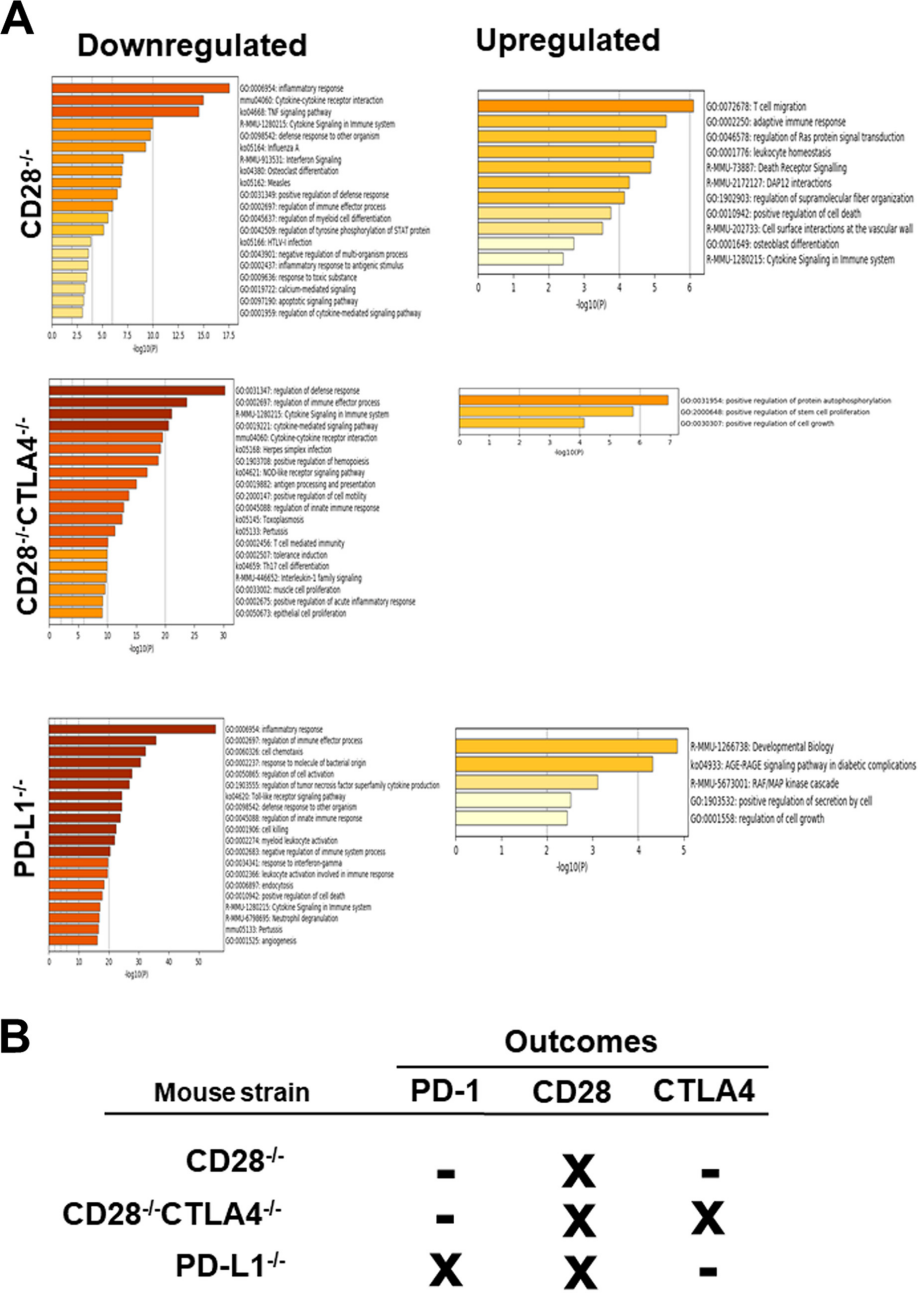
Enrichment analysis of gene ontology pathways for upregulated and downregulated genes in latently infected TG from CD28^−/−^, CD28^−/−^ CTLA4^−/−^, and PD-L1^−/−^ mice. Following NanoString analysis (as described for Fig. 5), we stratified normalized data based on fold expression over WT that are statistically significant (*P* < 0.05). (A) Upregulated and downregulated pathways for CD28^−/−^, CD28^−/−^ CTLA4^−/−^, and PD-L1^−/−^ infected mice TG are shown. (B) An overview of the net effect due to the absence of PD-L1, CD28, or CD28-CTLA4 on expression of PD-1, CD28, and CTLA4 in each group of CD28^−/−^, CD28^−/−^ CTLA4^−/−^ and PD-L1^−/−^ infected mice TG are shown. The minus sign represents inhibitory immune function, while X represents a blocked pathway.

The upregulated and downregulated genes in each knockout group were analyzed to determine their associations with relevant genetic pathways (Fig. 6A). In the downregulated group of genes for CD28^−/−^ mice, the top three enriched gene ontology pathways significantly affected, in order of greatest statistical significance, were inflammatory response (GO:0006954), cytokine-cytokine receptor interaction (mmu04060), and tumor necrosis factor (TNF) signaling pathway (ko04668) (Fig. 6A, CD28^−/−^, downregulated). The top upregulated pathways in CD28^−/−^ mice were T cell migration (GO:0072678) and adaptive immune response (GO:0002250) (Fig. 6A, CD28^−/−^, upregulated). The top downregulated pathways in CD28^−/−^ CTLA4^−/−^ mice were regulation of defense response (GO:0031347), regulation of immune effector process (GO:0002697), cytokine signaling (R-MMU-1280215), and herpes simplex infection (Ko05168) pathways (Fig. 6A, CD28^−/−^ CTLA4^−/−^, downregulated), while the positive regulation of the kinase autophosphorylation pathway (GO:0031954) was upregulated (Fig. 6A, CD28^−/−^ CTLA4^−/−^, upregulated). Finally, the top downregulated pathways in PD-L1^−/−^ infected mice were inflammatory responses (GO:0006954), regulation of immune effector process (GO:0002697), and cell chemotaxis (GO:0060326) (Fig. 6A, PD-L1^−/−^, downregulated), while the raf/map kinase cascade (R-MMU-5673001) and developmental biology-related pathways (R-MMU-1266738) were upregulated (Fig. 6A, PD-L1^−/−^, upregulated).

The expression of PD-1, CD28, and CTLA4 in the mouse strains PD-L1^−/−^, CD28^−/−^, and CD28^−/−^ CTLA4^−/−^ is summarized in Fig. 6B. Our results suggest that the loss of CD28 blocked CD28 downstream signaling while PD-1 or CTLA4 signaling was not affected (Fig. 6B). In CD28^−/−^ CTLA4^−/−^ mice, CD28 and CTLA4 expression were both affected, but their absence did not affect PD-1 expression. In contrast, loss of PD-L1 affected not only PD-1 expression but also CD28 expression. Taken together, these signaling pathways orchestrate complex immune responses that are modulated by multiple costimulatory molecules.

### Effect of the absence of CD28, CTLA4, and PD-L1 genes on IFNs and exhaustion markers in TG of latently infected mice.

**FIG 7:**
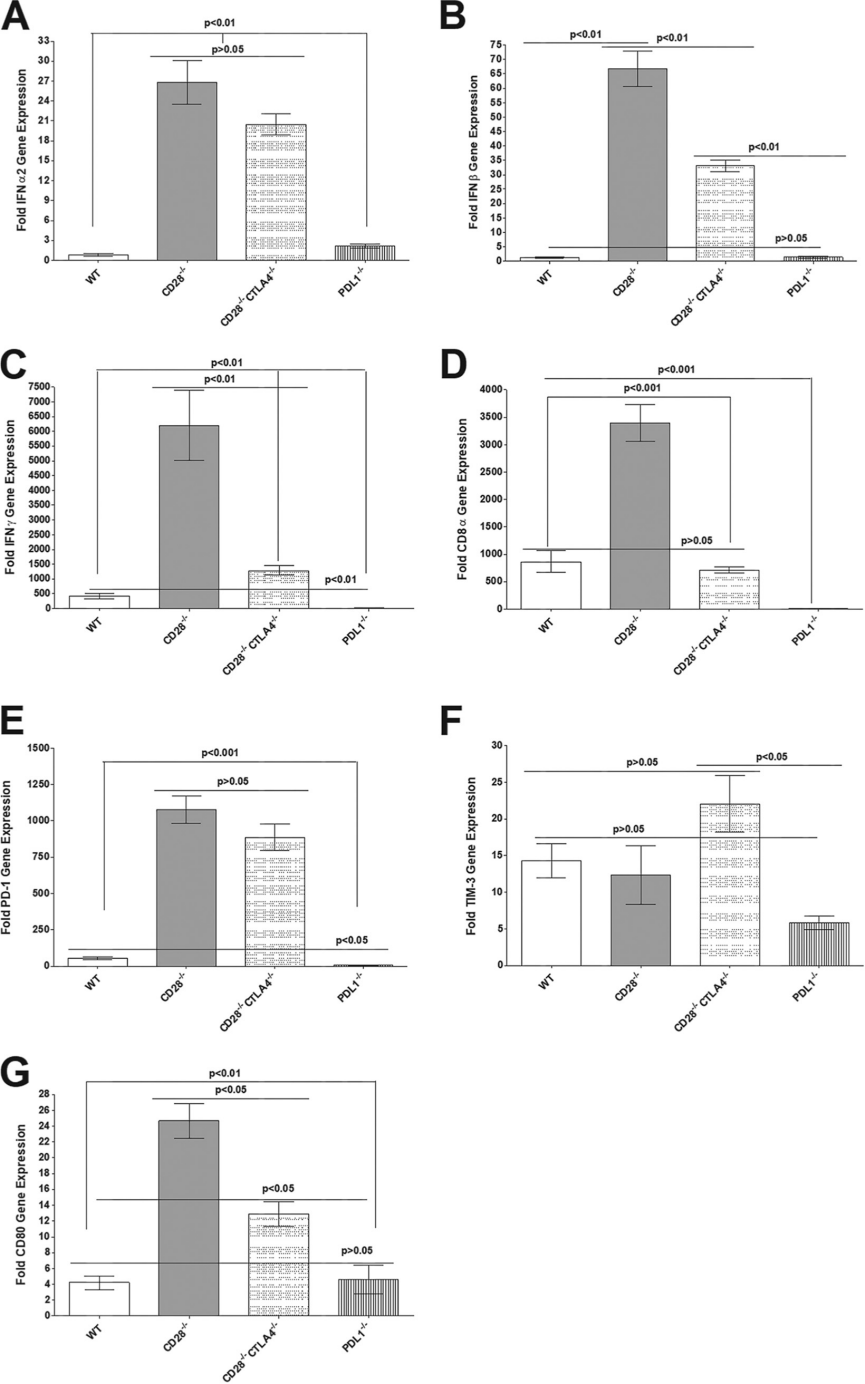
Quantitation of IFN-α2, IFN-β, IFN-γ, CD8, PD-1, Tim-3, and CD80 RNA transcripts in TG of latently infected mice. Corneas from CD28^−/−^, CD28^−/−^ CTLA4^−/−^, PD-L1^−/−^, and WT mice were scarified before ocular infection and infected ocularly with 2 × 10^5^ PFU per eye of KOS virus (as described for Fig. 1). On day 28 p.i., TG from infected mice were harvested, and qRT-PCR was performed on individual mouse TG. Expression of IFN-α2, IFN-β, IFN-γ, CD8, PD-1, Tim-3, and CD80 transcripts in TG of each infected mouse strain was normalized to the level of each transcript in the TG of their corresponding uninfected mouse strain (shown as fold increase or decrease compared to levels in their uninfected counterpart). GAPDH expression was used to normalize expression of each transcript in the TG of ocularly infected mice. Each bar represents the means ± SEM from CD28^−/−^ infected mice (20 TG), CD28^−/−^ CTLA4^−/−^ infected mice (18 TG), PD-L1^−/−^ infected mice (22 TG), and WT infected mice (12 TG). (A) IFN-α2 transcript; (B) IFN-β transcript; (C) IFN-γ transcript; (D) CD8 transcript; (E) PD-1 transcript; (F) Tim-3 transcript; (E) CD80 transcript.

Previously, we have shown a direct relationship between latency levels and increased exhaustion markers in TG of latently infected WT mice (21, 27, 29). Here, we investigated the effects of CD28, CTLA4, or PD-L1 absence on IFN expression, T cell exhaustion, and CD80 expression in TG of latently infected mice. Relative levels of IFN-α, IFN-β, IFN-γ, CD8, PD-1, Tim-3, and CD80 transcripts were determined in TG of latently infected CD28^−/−^, CD28^−/−^ CTLA4^−/−^, PD-L1^−/−^, and WT control mice by RT-PCR of total TG RNA extracts (Fig. 7). The results are presented as fold increase in infected CD28^−/−^, CD28^−/−^ CTLA4^−/−^, PD-L1^−/−^, and WT mice compared to baseline mRNA levels in TG from their uninfected naive counterparts. Levels of IFN-α in CD28^−/−^ and CD28^−/−^ CTLA4^−/−^ infected mice were similar (Fig. 7A) (*P*  > 0.05) and significantly higher than those in WT and PD-L1^−/−^ mice (Fig. 7A) (*P*  < 0.01). Similarly, the level of IFN-α was also significantly higher in PD-L1^−/−^ mice than in WT mice (Fig. 7A) (*P*  < 0.01).

IFN-β levels were significantly higher in CD28^−/−^ infected mice than in CD28^−/−^ CTLA4^−/−^ infected mice (Fig. 7B) (*P* < 0.01), and both were significantly higher than in WT and PD-L1^−/−^ infected mice (Fig. 7B) (*P*  < 0.01), while IFN-β expression in WT and PD-L1^−/−^ infected mice was the same (Fig. 7B) (*P*  > 0.05). IFN-γ levels in CD28^−/−^ infected mice were significantly higher than those in other infected mice (Fig. 7C) (*P*  < 0.01). Similarly, IFN-γ levels in CD28^−/−^ CTLA4^−/−^ mice were significantly higher than those in WT and PD-L1^−/−^ infected mice (Fig. 7C) (*P*  < 0.01), while PD-L1^−/−^ infected mice had lower IFN-γ levels than WT mice (Fig. 7C) (*P*  < 0.01). CD8α mRNA in the TG of latently infected CD28^−/−^ mice was significantly higher than levels in WT, CD28^−/−^ CTLA4^−/−^, and PD-L1^−/−^ infected counterparts (Fig. 7D) (*P* < 0.001). However, levels of CD8α mRNA in WT and CD28^−/−^ CTLA4^−/−^ infected mice were similar (Fig. 7D) (*P* > 0.05), and PD-L1^−/−^ infected mice had the lowest levels of CD8α mRNA. PD-1 mRNA levels in the TG of the latently infected CD28^−/−^ and CD28^−/−^ CTLA4^−/−^ mice were similar (Fig. 7E) (*P* > 0.05), and both were significantly higher than levels in WT and PD-L1^−/−^ infected counterparts (Fig. 7E) (*P* < 0.001). Finally, levels of PD-1 mRNA in WT mice were also higher than those in infected PD-L1^−/−^ mice (Fig. 7E) (*P* < 0.05). Tim-3, similar to PD-1, is considered an exhaustion marker; however, no significant differences were detected between WT, CD28^−/−^, and CD28^−/−^ CTLA4^−/−^ infected TG (Fig. 7F) (*P* > 0.05) or between WT and CD28^−/−^ groups with the PD-L1^−/−^ group (Fig. 7F) (*P* > 0.05). However, TG from infected CD28^−/−^ CTLA4^−/−^ mice had significantly higher levels of Tim-3 mRNA than TG from PD-L1^−/−^ infected mice (Fig. 7F) (*P* < 0.05). The absence of binding CD28 or both CD28 and CTLA4 in CD28^−/−^ and CD28^−/−^ CTLA4^−/−^ mice, respectively, produced a significant increase in CD80 mRNAs in TG of infected mice compared with WT and PD-L1^−/−^ mice (Fig. 7G) (*P* < 0.05). CD28^−/−^ mice also had higher levels of CD80 mRNA expression in their TG than did CD28^−/−^ CTLA4^−/−^ mice (Fig. 7G) (*P* < 0.05), and CD80 mRNA levels in PD-L1^−/−^ mice were similar to that of WT mice (Fig. 7G) (*P* > 0.05).

In summary, the absence of CD28 and CTLA4 in both CD28^−/−^ and CD28^−/−^ CTLA4^−/−^ infected mice correlated with higher expression of IFN-α, IFN-β, IFN-γ, PD-1, and CD80 transcripts, while higher expression of CD8 mRNA correlated with the absence of CD28. These results show that lower reactivation directly correlates with higher expression of IFNs and PD-1 transcripts.

## DISCUSSION

The major characteristic of HSV infection is its ability to establish latency in neurons of an infected host (30–32). Once acquired, latent infection demonstrates a lifelong pattern of episodic recurrence (33–35) in which the virus reactivates and travels back to the original site of infection, causing recurrent disease (22, 23). Due to the preexisting immune response, herpes stromal keratitis (HSK), also known as corneal scarring (CS), is much more likely to occur following recurrent HSV infection than a primary infection (36). Despite considerable efforts, neither a vaccine to prevent ocular infection nor safe and effective approaches to eliminate latent infections have been developed. Recently, we have shown that the ICP22 gene of HSV-1 binds to CD80 and suppresses CD80 but not CD86 expression in the cornea of infected mice (1). We have also shown that a recombinant HSV-1 lacking the ICP22 gene can exacerbate eye disease in ocularly infected mice while its parental control WT virus does not, despite the fact that the mutant virus replicates less efficiently *in vitro* and *in vivo* (3). In addition, overexpression of CD80 exacerbates corneal scarring in ocularly infected mice compared with parental control virus (4).

**FIG 8:**
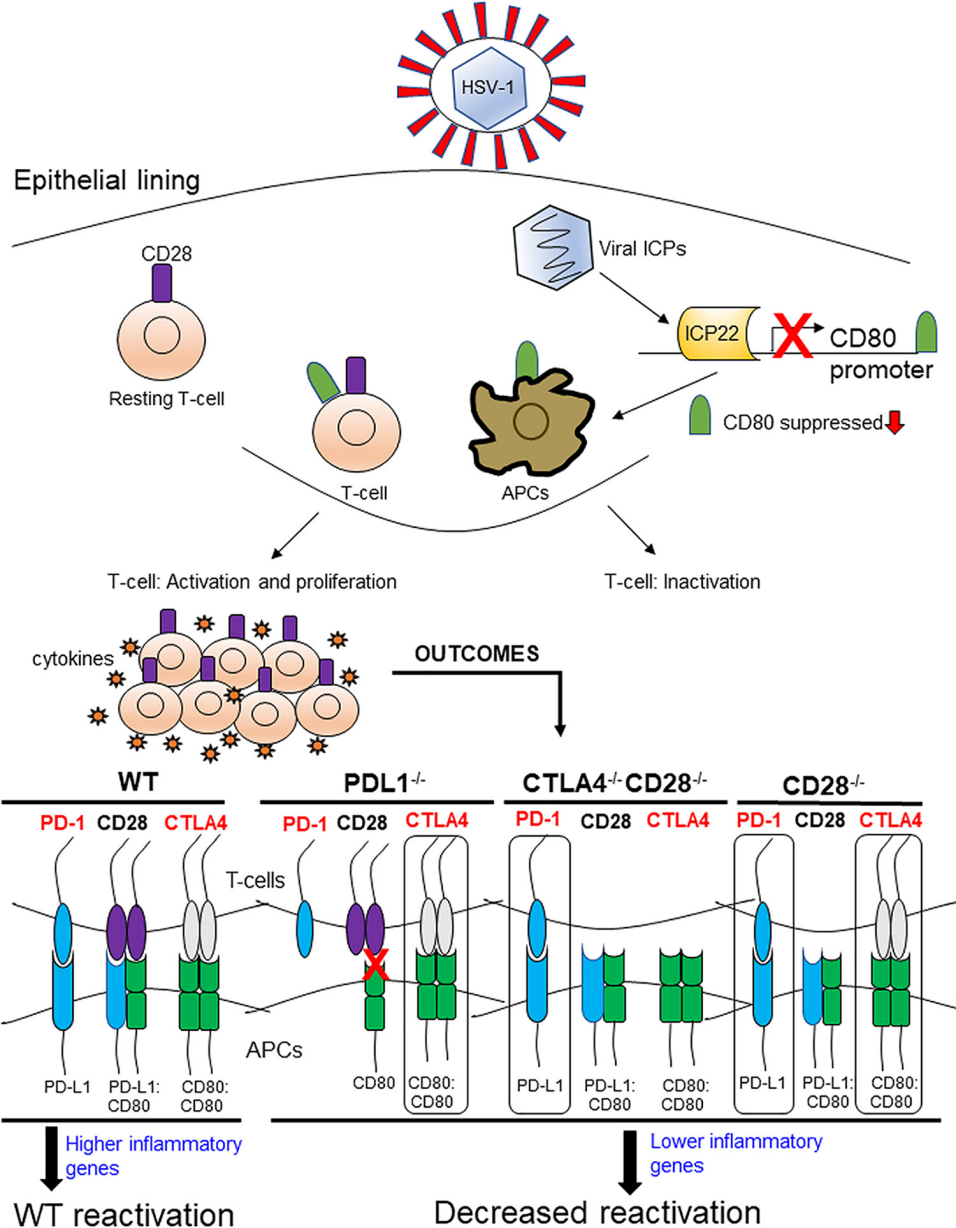
Graphical overview: absence of PD-L1, CTLA4, and CD28 in latently infected mice leads to decreased reactivation. HSV-1 gains entry through various routes in the epithelial lining of the eye. Once in the cell, the viral capsid is transported to the nucleus, where it rapidly undergoes viral gene replication. The first set of genes initiated for replication is 5 immediate-early genes encoding infected cell protein ICP0, ICP4, ICP22, ICP27, and ICP47. During this process, ICP22 is expressed in innate immune cells, such as APCs, and suppresses CD80 expression. The progeny virus can alert innate immune cells like naive T cells that activate PRR (pathogen-recognition receptors), inducing antiviral activities and host immune defenses. In APCs a similar process is taking place, where PRR mediated signaling activates APCs to upregulate MHC class II molecules and costimulatory molecules as well as proinflammatory cytokines. Release of IFN type I cytokines drives expansion and proliferation of activated T cells. In some cases, without appropriate costimulation, T cells can undergo inactivation, a process of apoptosis that usually occurs after an infection has resolved. In this study, WT mice were compared to PD-L1^−/−^, CD28^−/−^, and CTLA4^−/−^ CD28^−/−^ mice, each lacking specific immune receptors that block their binding to CD80. The immune ligands are the following: PD-L1 binds PD-1, the heterodimer PDL1:CD80 activates CD28, and the homodimer CD80:CD80 activates CTLA4. Loss of PD-L1 blocked both CD28- and PD-1-mediated activation, while the CTLA4 receptor was not affected. In CTLA4^−/−^ CD28^−/−^ mice both receptors are absent, leaving PD-L1/PD-1 signaling to predominate in T cells. Finally, the loss of CD28 left both PD-1 and CTLA4 to mediate immune signaling. Our study uncovered that the absence of PD-L1, CD28, or CTLA4 during latency reduced the number of reactivated TGs, which correlated with lower expression of inflammatory genes.

It is now clear that costimulatory molecules play a crucial role in regulating T cell activation, differentiation, effector function, and survival (8–11). Consistent with our results regarding the role of CD80 and ICP22 interaction in HSV-1 infection, it is not known whether costimulatory molecules affect CD80 function and, thus, HSV-1 infectivity. The function of CD80, a member of the immunoglobulin superfamily, depends on its binding to CD28, CTLA4, and PD-L1 (8–11, 14, 15). Since our previous studies ruled out the importance of CD86 in our model of ocular HSV-1 infection (1), this study extends those findings by showing a pathological role of CD80 in eye disease (1, 3) and further explores the importance of CD28, CTLA4, and PD-L1 binding to CD80 in HSV-1-induced immunopathology. Thus, in this study, we evaluated what role the absence of CD28, CTLA4, and PD-L1 binding to CD80 may play in HSV-1 primary infection, eye disease, and latency reactivation using CD28^−/−^, CD28^−/−^ CTLA4^−/−^, and PD-L1^−/−^ mice. Because CTLA4^−/−^ mice are not viable, we used CD28^−/−^ CTLA4^−/−^ mice that are viable; however, our results suggest that the phenotype of CD28^−/−^ mice is different from that of CD28^−/−^ CTLA4^−/−^ mice, confirming different functions of CD28 and CTLA4. A summary of the results obtained here with regard to the role of costimulatory molecules in HSV-1 reactivation is presented schematically in Fig. 8.

In this study, we observed higher virus replication in the eyes of CD28^−/−^, CD28^−/−^ CTLA4^−/−^, and PD-L1^−/−^ mice than in WT control mice, and this higher virus replication correlates with higher latency. We also showed that higher latency in CD28^−/−^ and CD28^−/−^ CTLA4^−/−^ mice correlated with higher PD-1 expression, which is consistent with our previous report that higher LAT RNA in the TG of mice latently (20, 37, 38) infected with wild-type HSV-1 strain McKrae correlated with higher PD-1 mRNA expression (21, 29). Previous studies have shown that chronic HSV-1 infection causes T cell exhaustion (20, 37, 38). Similar to our previous study showing that the absence of PD-L1 but not PD-L2 affected levels of latency and exhaustion (21), here we found that PD-L1^−/−^ mice also had lower levels of PD-1 expression. Our published reports have shown that upregulation of Tim-3 (T cell immunoglobulin and mucin domain-containing protein-3) correlated with T cell exhaustion (21). However, here we found that in contrast to PD-1 expression, higher latency in TG of CD28^−/−^, CD28^−/−^ CTLA4^−/−^, and PD-L1^−/−^ infected mice compared with WT mice did not correlate with higher Tim-3 expression. Multiple genes are implicated in T cell exhaustion, but based on our current and previous studies, PD-1 is the more relevant indicator of T cell exhaustion.

In this study, we looked at expression of gB, gK, and ICP22 transcripts during latency using a NanoString system. In contrast to higher expression of LAT in the three knockout mice compared to WT mice, ICP22 and gB expression levels decreased by 3- and 2.3-fold, respectively, in the three knockout mice compared to WT mice, while the level of gK expression was similar between the three knockout mice and WT mice. Thus, lower expression of ICP22 and gB in the three knockout mice correlated with lower reactivation. These results are similar to those of our published study in which blocking of ICP22 binding to CD80 delayed virus clearance (1) and a recombinant HSV-1 lacking ICP22 had less viral replication, less latency, and less reactivation than WT control virus (3). In line with significantly lower levels of ICP22 in the three knockout mice than in WT control mice, ICP22 deletion is known to significantly reduce viral replication and decrease its ability to establish latent infection in TG of HSV-1-infected mice (39). Thus, the absence of CD80 binding to CD28, CTLA4, or PD-L1 resulted in significant reduction of ICP22 transcripts and may have contributed to significant reduction in reactivation rates in infected CD28^−/−^, CD28^−/−^ CTLA4^−/−^, and PD-L1^−/−^ mice compared with WT mice. Thus, we hypothesized that binding of CD80 to its ligands as well as to ICP22 would result in faster reactivation and more eye disease. This is in line with our previous study showing that a recombinant HSV-1-expressing CD80 caused more eye disease in ocularly infected mice (1). HSV-1 infection is characterized by its ability to establish latency in the ganglia of infected individuals (31). The pattern of latency is similar in human and animals (31, 40, 41). Reactivation from latency is also a hallmark of HSV-1 infection, and this reactivation is a major cause of eye disease (22). In this study, we have shown that blockade of these costimulatory factors reduces reactivation, which may have therapeutic potential to control reactivation and reduce eye disease associated with reactivation.

In this study, we also detected higher levels of IFN-α and IFN-β in TG of CD28^−/−^ and CD28^−/−^ CTLA4^−/−^ mice, which correlated with higher latency but slower reactivation. IFN-α/β play an important role in HSV infection *in vitro* and *in vivo*, and HSV-1 can evade IFN responses through several mechanisms (42–47). Several HSV-1 genes, including ICP27 (48), ICP0 (49), US11 (50), virion host shutoff (*vhs*) (51), US3 (52), and ICP34.5 (53), inhibit IFN-α/β signaling, while gH/gL activate IFN-α/β (54). Type II interferon (IFN-γ) has a minimal impact on acute virus replication but helps control reactivation either alone or by synergizing with type I interferons (IFN-α/β) (55). To determine if interferons played a role in this study, we measured IFN-α, IFN-β, and IFN-γ expression in TG of CD28^−/−^ and CD28^−/−^ CTLA4^−/−^ mice. Expression of IFN-α was similar in CD28^−/−^ and CD28^−/−^ CTLA4^−/−^ mice but higher in PD-L1^−/−^ mice. Expression of IFN-β and IFN-γ was higher in CD28^−/−^ and CD28^−/−^ CTLA4^−/−^ mice. We conclude that the absence of CD28 and CTLA4 is a crucial regulator of immune responses leading to higher IFN-α and IFN-β expression during latency, which results in delayed reactivation. Consistent with our results, exogenous IFN-β and IFN-γ have been shown to transiently suppress HSV reactivation in a neuron intrinsic manner (56), and the expression of many genes with known roles in antiviral defenses and IFN signaling (e.g., Mx1/2, STAT1/2, ISG15, IFIT2, IRF1/7/9, Daxx, PKR, TAP1, and USP18) is downregulated in three knockout mice strains used in our study (Table 2). Our finding that greater expression of IFN-β and IFN-γ in CD28^−/−^ and CD28^−/−^ CTLA4^−/−^ mice corresponds to reduced reactivation is consistent with the results of the above-mentioned study. To recap, our results lead us to hypothesize that blocking the interaction of CD28, CTLA4, and PDL1 to CD80 induces the expression of type 1 and type 2 interferons, which, in turn, suppress reactivation while maintaining latency.

In this study, we have shown that the absence of the crucial immune response regulators CD28 and CTLA4 leads to higher IFN-α and IFN-β expression during latency, which correlates with increased latency and higher CD8 T cell exhaustion. Thus, higher levels of IFN-α, IFN-β, and PD-1 correlated with higher latency but also with slower or no reactivation in the absence of the CD28 or CTLA4 immune check point. While the absence of PD-L1 contributed to slow or no reactivation, this delayed reactivation did not correlate with higher levels of IFN-α, IFN-β, or PD-1. Thus, slower reactivation in the absence of PD-L1 expression was different from the absence of CD28 and CTLA4 gene expression. In contrast to CD28, PD-L1 and CTLA4 are negative costimulatory molecules, and the absence of all three molecules despite their contradictory functions affects HSV-1 reactivation (5, 25, 57). Lower reactivation in the three knockout mice compared with WT mice could be due to a significant reduction in the expression of *Ccl12*, *Ccl2*, *Ccl7*, *Ccrl2*, *Cd25*, *Cd40*, *Cebpb*, *Clic4*, *Cxcl1*, *Cxcl10*, *Daxx*, *Fcgr1*, *Fcgr4*, *Ifit1bl1*, *Il1β*, *Il1rn*, *Irf2*, *Irf7*, *Isg15*, *Mx1*, *Mx2*, *Nampt*, *Socs1*, *Socs3*, *Tnfaip3*, and *Usp18* transcripts. These 26 genes are nearly evenly divided between five broad categories of (i) immune inflammation responses (7 genes), (ii) chemokines (6 genes), (iii) cytokines (5 genes), (iv) transcription factors (4 genes), and (v) antiviral responses (4 genes). Our results suggest that in contrast to CD28^−/−^, CD28^−/−^ CTLA4^−/−^, and PD-L1^−/−^ mice, in WT mice, these 26 genes plus HSV-1 ICP22 have been upregulated. Thus, the absence of CD28, CTLA4, or PD-L1 contributes to the significantly reduced expression of these 26 genes in TG of latently infected mice. Individually or together, these genes may have contributed to slower reactivation in TG of latently infected CD28^−/−^, CD28^−/−^ CTLA4^−/−^, and PD-L1^−/−^ mice. Our results showed that the three knockout mouse strains that lack binding to CD80 have a significantly slower *ex vivo* reactivation rate despite their increased viral titers during acute infection and increased viral LAT copy numbers compared to the WT during latency. Thus, the absence of interactions between CD28, CTLA4, or PDL1 with CD80 leads to increased viral replication, latency, and exhaustion but less reactivation as a result of reduced inflammation and cellular trafficking. Previously, we and others showed that LAT plays a major role in the level of latency and time of reactivation and that HSV-1 recombinant viruses lacking LAT have an approximately 3-fold reduction in the level of latency and an approximately 2-days-slower reactivation in infected mice and rabbits (21, 58). However, the absence of LAT did not completely block reactivation, as we have shown.

Our NanoString gene panel studies found fewer inflammation related cytokines and chemokines. CXCL10 is a ligand for the chemokine receptor CXCR3, which is highly expressed on activated T cells and is associated with recruitment of T cells to sites of inflammation. Downregulation of CXCL10 is a major indicator of impaired T cell migration to the site of inflammation (59). This could be further explained based on the enhanced T cell exhaustion (PD1) in TG of knockout mice in our study, which in turn interferes with T cell activation and function, as we have shown previously (21). CD28 expression is required for helper T cell polarization in response to infection (60), which agrees with our study in which all three knockout mice strains lack CD28 signaling. Another study showed that the absence of CD28 costimulation does not cause HSK but maintains latency in HSV-1-infected mice, similar to our current results (60). The absence of CD28, CTLA4, or PD-L1 in the three knockout mouse groups compared with WT mice did not affect eye disease despite the three knockout mouse groups having higher virus replication in their eyes than did WT control mice. This is probably due to lower inflammatory responses in the knockout mice than in WT mice. CD25 and CD40 are both important to initiate and establish inflammation. IRF2 and IRF7 are both involved in inflammation and play important roles in innate cells such as macrophages (61, 62), and CXCL1, CXCL10, CCL2, CCL7, and CCL12 are all involved in trafficking and establishing inflammation (63). Thus, significant reductions in the expression of these genes may contribute to more viral replication, enhanced latency, more T cell exhaustion, and less reactivation, which reflects reduced inflammation and cellular trafficking.

Taken together, our current findings confirm our previous results, which showed that the ICP22-CD80 interaction enhances HSV-1 infectivity and, thus, eye disease (1, 3). This study also extends our previous findings regarding the role of costimulatory factors in blocking reactivation. Thus, in this study we have shown that blockade of these costimulatory factors blocks reactivation and, therefore, may have a valuable therapeutic potential to control reactivation and reduce eye disease associated with reactivation.

## MATERIALS AND METHODS

### Virus and cells.

Rabbit skin (RS) cells were generated in our laboratory, prepared, grown in minimal essential medium (MEM) plus 5% fetal bovine serum (FBS), and used as described previously (58). Plaque-purified HSV-1 strain KOS, an avirulent HSV-1 strain, was grown in RS cell monolayers in MEM containing 10% fetal calf serum as described previously (64).

### Mice.

Inbred BALB/c mice were obtained from the Jackson Laboratory (Bar Harbor, ME). CD28^−/−^ and CD28^−/−^ CTLA4^−/−^ mice were obtained from the Mutant Mouse Regional Resource Center (University of California, Davis). PD-L1^−/−^ mice were a gift from Arlene Sharpe (Harvard University). All mice used in this study have a BALB/c background and were bred in-house. Both male and female (6- to 8-week-old) mice were used in the study. All animal procedures were performed in strict accordance with the Association for Research in Vision and Ophthalmology Statement for the Use of Animals in Ophthalmic and Vision Research and the NIH *Guide for the Care and Use of Laboratory Animals* (65). The animal research protocol was approved by the Institutional Animal Care and Use Committee of Cedars-Sinai Medical Center (protocols 5030 and 8837).

### Ocular infection.

CD28^−/−^, CD28^−/−^ CTLA4^−/−^, PD-L1^−/−^, and WT BALB/c mice are highly susceptible to ocular infection with virulent HSV-1 strain McKrae even at a 100-fold lower dose of virus; thus, in this study, we used the avirulent HSV-1 strain KOS for infection. CD28^−/−^, CD28^−/−^ CTLA4^−/−^, PD-L1^−/−^, and WT BALB/c mice were infected ocularly with 2 μl of tissue culture media containing 2 × 10^5^ PFU/eye of the HSV-1 strains KOS with corneal scarification as we described previously (3). Before corneal scarification and ocular infection, mice were anesthetized with ketamine plus dexmedetomidine. Upon induction of anesthesia and ocular infection, buprenorphine was administered via subcutaneous injection. Buprenorphine was administered again the morning after infection.

### Detection of virus in tears of infected mice.

Tear films were collected from both eyes of 20 mice per group on days 1 to 7 p.i. using a Dacron-tipped swab (66). Each swab was placed in 1 ml of tissue culture medium and squeezed, and the amount of virus was determined using a standard plaque assay on RS cells.

### Monitoring CS.

HSV-induced corneal scarring (epithelial keratitis) in the corneas of infected mice was examined by slit-lamp biomicroscopy on day 28 p.i. Scoring was the following: 0, normal cornea; 1, mild haze; 2, moderate opacity; 3, severe corneal opacity but iris visible; and 4, corneal rupture and necrotizing keratitis. Each cornea was examined, and the means ± standard errors of the means (SEM) were calculated for each group.

### NanoString gene expression analysis of latent infected TG.

WT, CD28^−/−^, CD28^−/−^ CTLA4^−/−^, and PD-L1^−/−^ mice were ocularly infected with 2 × 10^5^ PFU/eye of HSV-1 strain KOS after corneal scarification. TG from infected mice on day 28 p.i. were isolated, and total RNA from individual TG was isolated. RNA samples for use in the nCounter sample input were pooled from three mice (6 TG), and each experiment was repeated three times using TG from nine mice per strain of mouse. The total RNA concentration for each well remained at 20 ng/μl. All hybridizations were performed in a total volume of 18 μl hybridization cocktail (3 μl of reporter Codeset, 2 μl of Reporter plus, 5 μl of hybridization buffer, 3 μl of capture mix, and 5 μl of sample) and mixed and centrifuged as described by the manufacturer (NanoString Tech, Seattle, WA). Reactions were processed in a thermocycler at 65°C for 20 h. Samples in a 12-well PCR strip were loaded into the MAX/FLEX nCounter Prep and loaded with consumables, such as reagent plates and a cartridge for the hybridization to take place. The preparation station ran for 3 h. The cartridge was transferred to the Digital Analyzer (NanoString Tech, Seattle Washington) for imaging analysis. A field of view of 240 was used for the experiment, and the Digital Analyzer ran for an additional 3 h for each cartridge.

For NanoString gene expression analysis, the gene panel nCounter MM Myeloid Innate Immunity V2 panel system was used (catalog number XT-CSO-MM112-12), which contains probes for 754 genes, including 264 genes associated with dendritic cells, 137 genes associated with neutrophils, 332 genes associated with macrophages, and 20 internal reference genes (8 negative, 6 positive, and 6 housekeeping) for data normalization. In addition to the 754 genes used, we customized the panel to include 10 more gene probes (*CD4*, *CD8*, *Foxp3*, *Gzmβ*, *CD25*, *CD103 (ITGAE)*, *HSV-1 gB*, *HSV-1 ICP22*, *HSV-1 gK*, and *Prf1*) that included HSV-1 markers and additional inflammatory and lymphocyte T cell markers not included in the original mouse myeloid panel. RNA from harvested HSV-1-infected TG was purified with RNeasy (Qiagen) as previously described and analyzed using the nCounter platform. The nSolver software 4.0 was used to analyze NanoString gene expression values and for principal components of probe counts, fold change, heatmap, and pathway analysis (Metascape). Metascape was used to identify the pathways or biochemical complexes enriched within this data set. This web-based portal simplifies generating relevant comprehensive gene list annotations and memberships into biochemical functions and genomic pathways, including functional enrichment analysis for the analysis and interpretation of genomics-based studies.

### RNA extraction, cDNA synthesis, and TaqMan RT-PCR.

TG from individual mice were collected on day 28 p.i., immersed in RNAlater RNA stabilization reagent (Thermo Fisher Scientific, Waltham, MA), and stored at −80°C until processing. In some experiments, TG from uninfected mice of the same age as infected mice were collected as described above. Total RNA extraction was conducted as we have described previously (67, 68). Levels of LAT RNA from latent TG were determined using a custom-made primer and probe set for LAT: forward primer, 5′-GGGTGGGCTCGTGTTACAG-3′; reverse primer, 5′-GGACGGGTAAGTAACAGAGTCTCTA-3′; probe, 5′-6-carboxyfluorescein-ACACCAGCCCGTTCTTT-3′ (amplicon length, 81 bp). The amplicon for the LAT primer set corresponds to LAT nucleotides (nt) 119553 to 119634. Relative LAT copy numbers were calculated using standard curves generated from the plasmid pGem-LAT5317.

Levels of CD8, PD-1, Tim-3, IFN-γ, IFN-β, IFN-α2, and CD80 transcripts in TG were evaluated using commercially available TaqMan gene expression assays (Applied Biosystems, Foster City, CA) with optimized primer and probe concentrations. Primer probe sets consisted of two unlabeled PCR primers and the FAM dye-labeled TaqMan MGB probe formulated into a single mixture. Additionally, all cellular amplicons included an intron-exon junction to eliminate signals from genomic DNA contamination. The assays used in this study were the following: (i) CD8 (α chain), ABI Mn01182108_m1, amplicon length of 67 bp; (ii) PD-1 (programmed death 1; also known as CD279), ABI Mm00435532_m1, amplicon length of 65 bp; (iii) CD80, ABI MM00711660_m1, amplicon length of 117 bp; (iv) Tim-3, ABI Mm00454540_m1, amplicon size of 98 bp; (v) IFN-γ, ABI assay ID Mm00801778_m1, amplicon length of 101 bp; (vi) IFN-α2, ABI assay ID Mm00833961_s1, amplicon length of 158 bp; (vii) IFN-β1, ABI assay ID Mm00439552_s1, amplicon length of 69 bp; and (viii) glyceraldehyde-3-phosphate dehydrogenase (GAPDH) used for normalization of transcripts, ABI Mm999999.15_G1, amplicon length of 107 bp.

### Statistical analysis.

Protective parameters were plotted and analyzed for statistical significance using Student's *t* test, Fisher's exact test, one-way analysis of variance (ANOVA), or two-way ANOVA with Bonferroni posttest with GraphPad (GraphPad, San Diego, CA). Results were considered statistically significant if the *P* value was <0.05.
